# Exercise sustains motor function in Parkinson's disease: Evidence from 109 randomized controlled trials on over 4,600 patients

**DOI:** 10.3389/fnagi.2023.1071803

**Published:** 2023-02-14

**Authors:** Meiqi Zhang, Fang Li, Dongyu Wang, Xiaohong Ba, Zhan Liu

**Affiliations:** ^1^Department of Physical Education and Health Education, Springfield College, Springfield, MA, United States; ^2^Yale/VA Learning-Based Recovery Center, Yale University, New Haven, CT, United States; ^3^Department of Neurology, The First Affiliated Hospital of Jinzhou Medical University, Jinzhou, Liaoning, China; ^4^Department of Neurology, The Center Hospital of Jinzhou, Jinzhou, Liaoning, China

**Keywords:** exercise, Parkinson's disease, motor dysfunction, mobility, balance, manual dexterity

## Abstract

Physical exercise has been widely identified as a supplementary therapy for Parkinson's disease (PD). Evaluating changes in motor function over long-term periods of exercise and comparing efficacy of various exercise types will enable a better understanding of the effects of exercise on PD. In the current study, a total of 109 studies that covered 14 types of exercise were included in the analyses, enrolling 4,631 PD patients. The results of meta-regression revealed that chronic exercise delays the progression of PD motor symptoms, mobility, and balance decline deterioration, whereas for the non-exercise PD groups, motor function progressively decline. Results from network meta-analyses suggest that dancing is the optimal exercise for general motor symptoms of PD. Furthermore, Nordic walking is the most efficient exercise to mobility and balance performance. The results from network meta-analyses also suggest that Qigong may have specific benefit in improving hand function. The findings of the current study provide further evidence that chronic exercise preserves the progression of motor function decline in PD and suggest that dancing, yoga, multimodal training, Nordic walking, aquatic training, exercise gaming, and Qigong are effective PD exercises.

**Systematic review registration:**

https://www.crd.york.ac.uk/prospero/display_record.php?RecordID=276264, identifier: CRD42021276264.

## 1. Introduction

Motor deficits adversely affect the quality of life in people with Parkinson's disease (PD; Moustafa et al., 2016). Medical treatment of PD may reduce cardinal symptoms of PD (Lindenbach and Bishop, 2013), but it does not directly address motor functions associated with such a disease (Boelen, 2009). As a result, practitioners have recognized exercise as an alternative strategy in conjunction with medical treatments (Petzinger et al., 2013).

Researchers in previous systematic reviews and meta-analyses have selectively examined specific exercise modalities for motor functions of PD (e.g., Uhrbrand et al., 2015; Gomes Neto et al., 2020; de Oliveira et al., 2021). However, directly comparing more than two types of exercise using conventional pairwise meta-analysis has some major limitations. For example, the key elements of an exercise modality remain unclear, which may affect the implementation of effective interventions. There has been one network meta-analysis studying focusing on comparison of eight types of exercises for patients with PD (Tang et al., 2019). In an attempt to advance our understanding of the effects of exercise modalities on motor functions of PD, researchers in the current study included more exercise types in the review and added manual dexterity as an outcome variable to the analysis.

Further, the long-term beneficial effects of participation in exercise were rarely examined and discussed. A meta-analysis by Shen et al. (2016) examined the effects of exercise on balance and gait in PD by categorizing intervention duration as short-term and long-term. To better understand the relatively long-term effects of exercise on motor functions of PD, additional quantitative evidence would be more clinically meaningful. Meta-regressions analysis may be a potentially effective approach to obtaining additional quantitative evidence of long-term effects of exercise on motor functions of PD if such analysis follows the progression of general motor symptoms [the Unified Parkinson's Disease Rating Scale (UPDRS) - motor examination], balance, and mobility over the duration of exercise treatments.

The aims of the study were therefore to (a) investigate the quantified long-term changes in motor function of PD which are specifically due to exercise over time, and (b) compare the efficacy of various exercises for motor function of PD.

## 2. Methods

The systematic review protocol was developed using guidance from Preferred Reporting Items for Systematic Reviews and Meta-Analyses (PRISMA) statement (Page et al., 2021), and registered with PROSPERO (CRD42021276264).

### 2.1. Search strategy and selection criteria

We performed a comprehensive literature search for published randomized clinical trials (RCTs) in Web of Science, PubMed, Embase, and MEDLINE from inception to August 2021 (see search strategy in the Supplementary material). Only studies published in English were considered. All the inclusion criteria followed the Patients, Interventions, Comparisons, Outcomes, and Study design framework (Hutton et al., 2015). The population group of interest was adults (≥18 years old) with clinically diagnosed Parkinson's disease. Eligible interventions included any types of exercise treatment for motor functions of patients with PD, without the addition of other therapies (other than regular medication) for at least 4 weeks/8 h of intervention duration. A minimum of two trials were needed to assess an intervention type to be included in the meta-analyses. Potential comparators were true control and active control (Table 1). Studies were required to include at least one of the outcome measures of interest: UPDRS motor scale, balance performance (e.g., Berg balance scale, BBS), mobility performance (e.g., timed up-to-go test, TUG), or manual dexterity (e.g., Purdue pegboard test). If multiple tools were used to assess a certain outcome, the most appropriate one was selected according to the prescribed hierarchy.

**Table 1:** Definitions of exercise interventions and controls.

**Type**	**Definition**
True control	No intervention provided
Active control	Treatment including education, psychological, or other non-physical interventions
Aquatic exercise	Exercise performed in water
Boxing	Exercise following boxing techniques such as punches and defenses
Cycling	Exercise using a special stationary exercise bicycle with a weighted flywheel in an indoor setting
Dancing	Exercise form consisting of sequences of movement, either improvised or purposefully selected
Exergaming	Exercise performed by video games
Functional	Exercise targeting on train muscles to work together and prepare them for daily tasks
Multimodal	Two or more of specific types of exercise
Nordic walking	Walking exercise performed with specially designed walking poles like ski poles
Ordinary walking	Walking exercise practicing by using treadmill or land-based modes
Qigong	Exercise typically focusing on movement, breathing, and meditation, to integrate body, breath, and mind adjustments into one
Strength training	Exercise designed to improve muscular strength, power, and endurance
Stretching	Exercise consisting of muscle flexing or lengthening only
Tai chi	Exercise following traditional Chinese martial art principles to improve strength, balance, and physical function
Yoga	Exercise following the principles of traditional yoga with physical components

### 2.2. Data extraction

Study characteristics (e.g., author, year, title, journal), study design (e.g., two-or multiple-arm trial), number of patients, patient characteristics (e.g., age, gender, duration of PD, H&Y stage, body mass index, UPDRS total score, and UPDRS motor score), interventions (e.g., type, frequency, intervention duration, and session duration), and outcomes (e.g., UPDRS motor score, balance performance, mobility performance, and manual dexterity) were extracted by the two reviewers. Extracted outcome data were pre- and post-test mean, standard deviation (SD), and sample size. Data presented as medians, ranges, intervals were converted to mean and SD.

### 2.3. Risk of bias assessment

The Cochrane Collaboration's Tool for Assessing Risk of Bias was used to evaluate the internal validity of results (Higgins and Green, 2008). The two reviewers examined six categories of bias, which included random sequence generation, allocation concealment, blinding of outcome assessment, incomplete data, selective outcome report, and other bias. Assessment items were rated “low risk,” “unclear,” or “high risk” based on the available information. As it was impossible to achieve patient blinding during exercise intervention, patient blinding was deemed as a high risk of bias and was not considered in the assessment process. Disagreement was resolved by discussion.

### 2.4. Statistical analysis

To investigate time effects of exercise interventions, bivariate linear correlations and a weighted linear meta-regression were conducted for the UPDRS motor scale, mobility, and balance measures. Despite low heterogeneity, only TUG (mobility assessment) and BBS (balance assessment) were included for the correlation analyses. For each study, the change score was calculated, which was the difference between baseline and posttest. Log transformations were applied for intervention duration data.

The comparative effectiveness was investigated using the network meta-analysis methodology of combining direct and indirect evidence for all treatment effects. First of all, the geometry of the network of evidence was summarized by using network plots (Shim et al., 2017). Secondly, consistency assumption was assessed by fitting both a consistency and inconsistency network meta-analysis, and the results of the Wald test (for global consistency) and side-splitting test (for local consistency) were considered. Thirdly, effect sizes were displayed by plots and league tables. Standardized mean difference (SMD; for primary and secondary outcomes)/mean difference (MD; especially for UPDRS subgroup analysis) and 95% confidence intervals (95% CIs) were displayed as effect estimates. Fourthly, the relative rankings of treatments were evaluated by surface under the cumulative ranking (SUCRA) and probability of being the best/worst treatment. Both the probabilities and SUCRA for estimating the cumulative ranking probabilities were reported, but SUCRA is considered more precise since it counts the ranking of all interventions that a given intervention is among the best treatments (Mbuagbaw et al., 2017; Shim et al., 2017). A SUCRA value of 100% indicates that this type of exercise intervention is certain to be the best in the network, whereas a value of 0 indicates that it is certain to be the least effective (Salanti et al., 2011). Finally, publication bias was checked by funnel plots.

In addition, pairwise random-effects analysis was implemented to examine the effects of each exercise treatment compared with the true control comparator. Effect sizes were estimated with standard mean differences (SMDs) and 95% confidence intervals (95% CIs) by the random effect size model. One-way analysis of variance (ANOVA) was also conducted to determine the differences of patient characteristics on exercise types. The F-ratio was used as a test for the null hypothesis of equality of the means.

Network meta-analyses were done by Stata 17.0 (StataCorp, 2021); Funnel plots and meta-regressions were generated by R packages “robvis (McGuinness and Higgins, 2021)” and “ggplot2 (Wickham, 2016)” in “R” statistical environment 4.1.0 (R-Core-Team, 2013).

## 3. Results

### 3.1. Study characteristics

**Figure 1:**
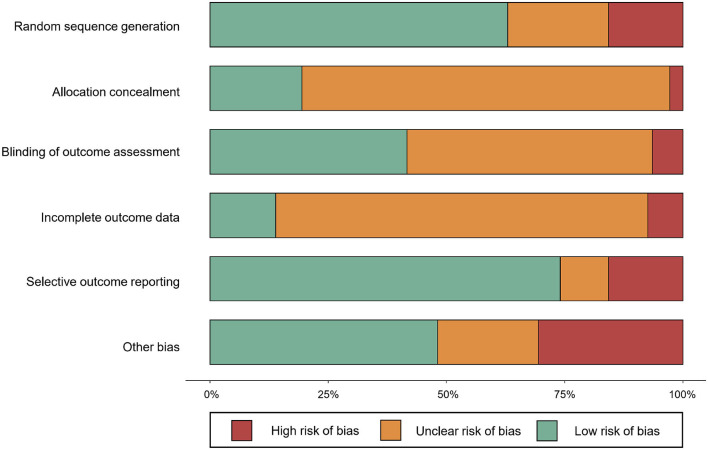
Risk of bias assessment of included randomized controlled trials.

A total of 4,185 records were identified after removing duplicates. Of the records, 620 were considered potentially relevant by screening titles and abstracts. A total of 109 articles (94 two-arm trials and 16 three-arm trials) were finally deemed eligible for the network meta-analysis (Supplementary Figure S1). The 109 studies covered 234 treatments, which were 176 exercise treatments and 58 non-exercise comparators. Exercise interventions included 14 modes of exercise: aquatic training (*n* = 14, 6.0%), boxing ( *n* = 2, 0.9%), cycling (*n* = 5, 2.1%), dancing (*n* = 19, 8.1%), exergaming (*n* = 12, 5.1%), functional training (*n* = 36, 15.4%), multimodal exercise (*n* = 15, 6.4%), Nordic walking (*n* = 6, 2.6%), Qigong (*n* = 6, 2.6%), strength training (*n* = 17, 7.3%), stretching (*n* = 2, 0.9%), Tai chi (*n* = 12, 5.1%), ordinary walking (*n* = 19, 8.1%), and yoga (*n* = 9, 3.9%, Table 1). Non-exercise treatments included true control (*n* = 13, 5.6%) and active control (*n* = 45, 19.2%; Table 1). No statistical difference was detected in patient characteristics by exercise modes: mean age, *F* = 1.040, *p* = 0.415; mean year of PD, *F* = 1.120, *p* = 0.342; and mean H&Y stage, *F* = 0.918, *p* = 0.550 (Supplementary Table S1). Detailed study characteristics and the list of included studies are reported in Supplementary Tables S2, S3, respectively. The risk of bias assessment for each study is summarized in Figure 1. Included studies tended to exhibit a relatively low risk of random sequence generation (60%) and selective outcome reporting (75%), but not allocation concealment (20%), blinding of outcome assessment (40%), incomplete outcome data (15%), and other bias (48%).

### 3.2. Motor changes over time

**Figure 2:**
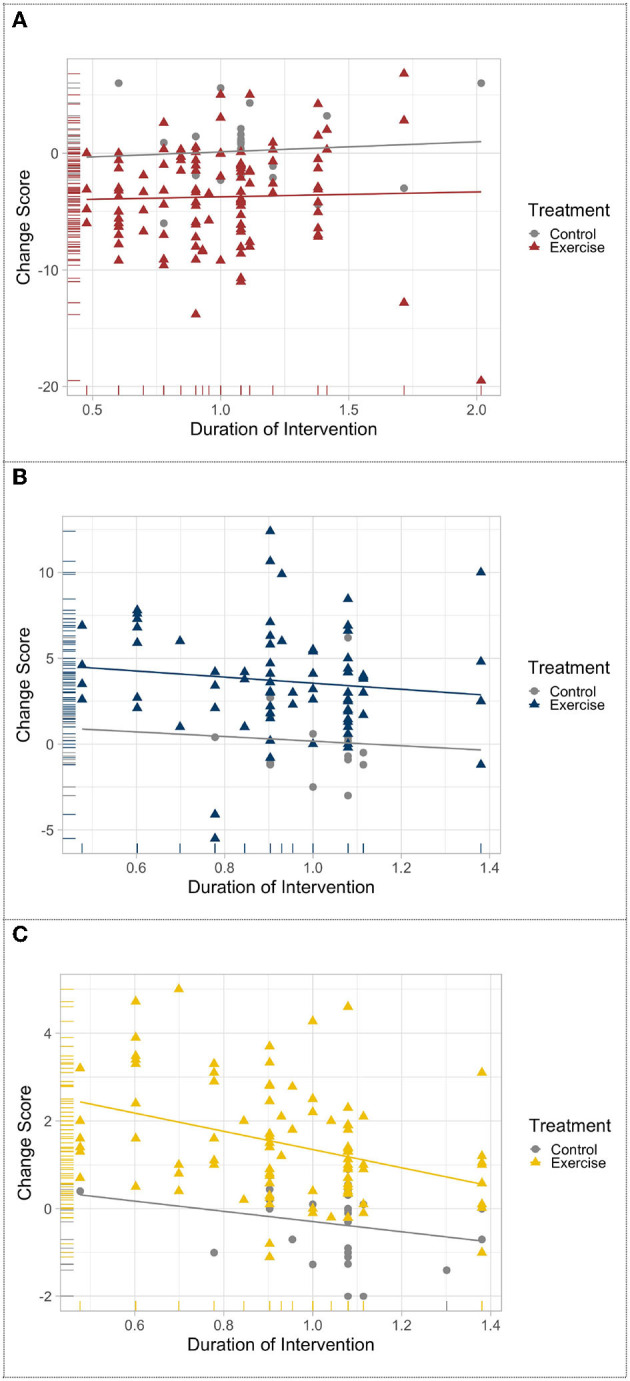
Meta-regression of the change in **(A)** UPDRS-motor scale, **(B)** balance, and **(C)** mobility after exercise treatment across time.

The results of meta-regression revealed that chronic exercise delays the progression of PD motor symptoms, mobility, and balance decline deterioration, whereas for the non-exercise PD groups, motor function progressively decline. Although slightly linear trends could be visually detected (Figures 2A, B), changes in UPDRS motor scale and Balance were not statistically associated with exercise intervention duration, *r* (109) = −0.029, *p* = 0.761, and *r* (76) = −0.123, *p* = 0.287. The change score of mobility was negatively related and significantly predictive of exercise intervention duration, *r* (97) = 0.204, *p* = 0.044, *R*^2^ = 0.145, and *F* (1.96) = 16.2554, *p* < 0.001 (Figure 2C).

### 3.3. Comparative effects of various exercise modalities

**Figure 3:**
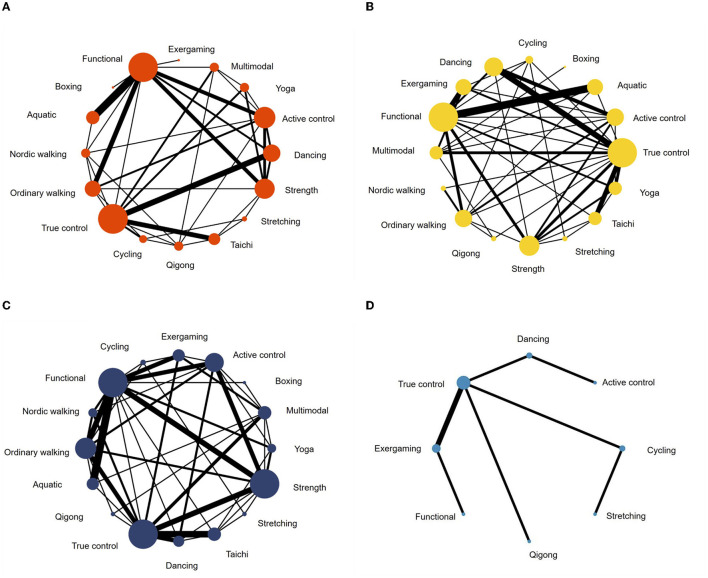
Network meta-analysis maps of studies examining the efficacy of exercise interventions in patients with Parkinson's disease on **(A)** UPDRS-motor scale, **(B)** balance, **(C)** mobility, and **(D)** manual dexterity.

A total of 62 studies with 2,668 patients were included in the network meta-analysis of the UPDRS—motor examination score (Figure 3A). Of the studies, 42 studies tested under “ON state” (i.e., good function), eight studies tested with “OFF state” (i.e., poor function), and 12 studies did not control/report medication state of testing. Testing for global inconsistency was not significant ($\chi_{28}^{2}$ = 31.92, *p* = 0.28). The test of local inconsistency from the node-splitting model showed a small percentage of inconsistency (3 of 37 comparison loops). The funnel plot did not provide evidence for apparent publication bias (Supplementary Figure S4A). The results of the consistency model indicated that dancing (*SUCRA*: 94%), yoga (*SUCRA*: 86%), multimodal training (*SUCRA*: 84%), aquatic training (*SUCRA*: 75%), and Nordic walking (*SUCRA*: 61%) were among the most effective interventions for UPDRS-motor examinations. Boxing (*SUCRA*: 2%), exergaming (*SUCRA*: 24%), and stretching (*SUCRA*: 12%) were most likely to be the least effective. Pairwise meta-analysis suggested that most exercise interventions were more effective than true control except for boxing, *SMD* = −0.96, 95% *CI* = −1.89 to −0.03, *p* = 0.043; stretching, *SMD* = −0.23, 95% *CI* = −0.72 to 0.27, *p* = 0.366, and exergaming, *SMD* = −0.12, 95% *CI* = −1.16 to 0.92, *p* = 0.817 (Table 2).

**Table 2:** Network meta-analysis consistency models for UPDRS-motor score, balance, mobility, and manual dexterity in studies examining the efficacy of exercise training in patients with Parkinson's disease.

	**Comparison to “true control”**		**Likelihood (%) of being**		
**Exercise mode**	**Pooled SMD (95% CI)**	***P-*** **value**	**Best**	**Worst**	**SUCRA (%)**
**UPDRS—Motor score**					
True control	**–**	**–**	0.0	0.3	19
Active control	0.38 (0.04 to 0.72)	**0.028**	0.0	0.0	49
Aquatic	0.62 (0.19 to 1.05)	**0.005**	4.9	0.0	75
Boxing	−0.96 (−1.89 to −0.03)	**0.043**	0.0	84.6	2
Cycling	0.04 (−0.39 to 0.48)	0.846	0.0	0.5	24
Dancing	0.89 (0.58 to 1.21)	**< 0.001**	47.3	0.0	94
Exergaming	−0.12 (−1.16 to 0.92)	0.817	1.8	8.9	24
Functional	0.41 (0.08 to 0.74)	**0.014**	0.0	0.0	51
Multimodal	0.74 (0.36 to 1.12)	**< 0.001**	14.2	0.0	84
Nordic walking	0.50 (0.05 to 0.95)	**0.029**	1.9	0.0	61
Ordinary walking	0.47 (0.10 to 0.84)	**0.012**	0.5	0.0	59
Qigong	0.46 (0.07 to 0.86)	**0.022**	0.8	0.0	59
Strength	0.47 (0.12 to 0.83)	**0.008**	0.1	0.0	60
Stretching	−0.23 (−0.72 to 0.27)	0.366	0.0	5.7	12
Tai chi	0.29 (−0.04 to 0.62)	0.080	0.0	0.0	42
Yoga	0.79 (0.31 to 1.28)	**0.001**	28.3	0.0	86
**Balance**					
True control	**–**	**–**	0.0	29.5	7
Active control	0.33 (0.00 to 0.65)	0.050	0.0	0.7	35
Aquatic	1.06 (0.72 to 1.41)	**< 0.001**	18.3	0.0	90
Boxing	−0.04 (−0.92 to 0.83)	0.922	0.3	49.6	16
Cycling	0.28 (−0.14 to 0.70)	0.184	0.0	2.6	32
Dancing	0.92 (0.62 to 1.21)	**< 0.001**	3.8	0.0	83
Exergaming	1.02 (0.69 to 1.35)	**< 0.001**	13.0	0.0	88
Functional	0.54 (0.27 to 0.82)	**< 0.001**	0.0	0.0	58
Multimodal	0.23 (−0.05 to 0.51)	0.113	0.0	0.9	26
Nordic walking	1.25 (0.63 to 1.88)	**< 0.001**	63.9	0.0	94
Ordinary walking	0.34 (0.01 to 0.67)	**0.045**	0.0	0.7	37
Qigong	0.32 (−0.12 to 0.76)	0.150	0.0	3.0	36
Strength	0.44 (0.15 to 0.73)	**0.003**	0.0	0.0	47
Stretching	0.16 (−0.28 to 0.60)	0.488	0.0	13.0	21
Tai chi	0.73 (0.42 to 1.04)	**< 0.001**	0.5	0.0	72
Yoga	0.55 (0.18 to 0.92)	**0.003**	0.1	0.1	57
**Mobility**					
True control	**–**	**–**	0.0	54.5	4
Active control	0.54 (0.12 to 0.96)	**0.012**	0.0	0.3	35
Aquatic	1.11 (0.65 to 1.57)	**< 0.001**	4.4	0.0	86
Boxing	0.20 (−0.66 to 1.06)	0.645	0.2	30.6	18
Cycling	0.62 (−0.07 to 1.32)	0.077	0.7	2.1	45
Dancing	1.07 (0.61 to 1.54)	**< 0.001**	4.3	0.0	83
Exergaming	0.81 (0.36 to 1.26)	**< 0.001**	0.1	0.0	64
Functional	0.75 (0.41 to 1.09)	**< 0.001**	0.0	0.0	58
Multimodal	0.62 (0.21 to 1.02)	**0.003**	0.0	1.1	43
Nordic walking	1.62 (1.09 to 2.16)	**< 0.001**	88.9	0.0	99
Ordinary walking	0.70 (0.32 to 1.07)	**< 0.001**	0.0	0.0	51
Qigong	0.64 (-0.01 to 1.30)	0.054	0.7	1.7	48
Strength	0.63 (0.29 to 0.98)	**< 0.001**	0.0	0.0	44
Stretching	0.40 (−0.34 to 1.13)	0.292	0.1	10.5	28
Tai chi	0.59 (0.19 to 0.99)	**0.004**	0.0	0.0	40
Yoga	0.75 (0.19 to 1.31)	**0.009**	0.0	0.1	56
**Manual dexterity**					
True control	**–**	**–**	0.3	0.0	49
Active control	−2.97 (−4.06 to −1.87)	**< 0.001**	0.0	89.3	2
Cycling	0.23 (−0.65 to 1.11)	0.608	13.6	0.0	68
Dancing	−2.51 (−3.30 to −1.71)	**< 0.001**	0.0	10.7	13
Exergaming	0.09 (−0.38 to 0.57)	0.698	2.6	0.0	57
Functional	0.26 (−0.73 to 1.25)	0.609	21.7	0.0	67
Qigong	0.58 (−0.11 to 1.28)	0.101	51.9	0.0	86
Stretching	0.12 (−0.86 to 1.11)	0.807	9.9	0.0	59

The bold values denote statistical significance at the *p* < 0.05 level.

A follow-up analysis of medication states revealed some inconsistent results. In terms of “ON” medication state (42 studies with 1,777 patients), dancing was still the most promising exercise mode (*SUCRA*: 89%), while aquatic training and ordinary walking had the slightly higher probability of being the most promising modes with *SUCRA* values of 85 and 82%, respectively (Supplementary Table S4). Considering the limited number of studies and the lack of direct comparisons for “OFF” state testing (8 studies with 379 patients), pairwise comparisons were applied (Supplementary Figure S3). The results showed that dancing, multimodal training, and ordinary walking were more effective than true control, whereas no significant difference was observed between aquatic training and functional training.

For balance performance, 71 studies with 2,771 patients were eligible to the network meta-analysis (Figure 3B). Testing for global inconsistency was significant ($\chi_{31}^{2}$ = 46.01, *p* = 0.040). The test of local inconsistency from the node-splitting model showed a small percentage of inconsistency (3 of 44 comparisons). No apparent evidence for publication bias was detected from the funnel plot (Supplementary Figure S4). The results of network meta-analysis suggested that Nordic walking (*SUCRA*: 94%), aquatic (*SUCRA*: 90%), exergaming (*SUCRA*: 88%), dancing (*SUCRA*: 83%), and Tai Chi (*SUCRA*: 72%) were most likely to be the most promising exercise modes to improve balance performance. True control (*SUCRA*: 7%) and boxing (*SUCRA*: 16%) were most likely to be the least effective. Pairwise meta-analyses demonstrated that, in comparison to the true control, most exercises (except boxing) improved balance performance, but stretching (*p* = 0.488), cycling (*p* = 0.184), Qigong (*p* = 0.150), and multimodal training (*p* = 0.113) were not statistically significant (Table 2).

For mobility performance, 78 studies (with 3,164 patients) were included in the network meta-analysis (Figure 3C). Testing for global inconsistency was not significant ($\chi_{39}^{2}$ = 27.70, *p* = 0.912). The test of local inconsistency from the node-splitting model showed a small percentage of inconsistency (3 of 48 comparisons). No apparent evidence for publication bias was detected from the funnel plot (Supplementary Figure S4C). The results of the consistency model indicated that Nordic walking (*SUCRA*: 99%) had the highest probability of being the best exercise treatment for improving mobility performance, followed by aquatic training (*SUCRA*:86%), dancing (*SUCRA*: 83%), exergaming (*SUCRA*: 64%), functional training (*SUCRA*: 58%), and yoga (*SUCRA*: 56%). True control (*SUCRA*: 4%) and boxing (*SUCRA*: 18%) were most likely to be the least effective. Pairwise comparisons showed that all the exercise intervention were more effective than true control, but no statistical difference was found in boxing (*p* = 0.645), stretching (*p* = 0.292), cycling (*p* = 0.077), and Qigong (*p* = 0.054; Table 2).

For manual dexterity, eight studies (with 392 patients) assessed manual dexterity, but network meta-analysis was relatively less objective since the limited types of treatments (Figure 3D). No apparent evidence for publication bias was detected from the funnel plot (Supplementary Figure S4D). Pairwise analysis showed that, compared with true control, most of the exercise interventions (5/6) slightly improved manual dexterity but not statistically significant. Dancing exercise intervention showed a significant negative effect in comparison to the true control (*p* < 0.001; Table 2).

## 4. Discussion

Researchers in the current study compared the effects of various exercise modes in sustaining motor function in PD. Evidence in this study was based on 234 treatments from 109 articles and included 4,631 PD patients. The current study was also designed to explore the efficacy of exercise for PD motor progression over time. Results from our study confirmed the optimal effects of dancing on overall motor function in patients with PD that reported by Tang et al. (2019). Moreover, findings in the present study suggested that, for patients with PD, dancing (for general motor symptoms), Nordic walking (for balance and mobility), and Qigong (for manual dexterity) might be the optimal exercise choices.

The meta-regression analysis maybe an effective approach to revealing the progression of motor symptoms in PD and examining the effects of chronic exercise on sustaining motor function. Significant group differences were observed in UPDRS-motor examination, balance, and mobility mean performance between exercise and non-exercise groups, which may represent the meaningful effects of participation in exercise for PD patients. Additionally, only the change in TUG performance showed a linear association with time suggesting a long-term beneficial effect of exercise on the mobility function of patients with PD. To some extent, such a finding may also suggest that the TUG is a more sensitive predictor to the progression of motor function in PD compared to UPDRS-motor scale and BBS.

As aforementioned, our network meta-analysis for general PD motor symptoms identified dancing as the exercise most likely to be the best for patients with PD. The underlying mechanism for this effect may be partially explained with the two factors. First, over the 14 exercise modes, dancing was the only one that applied rhythmic stimulation into practice. Rhythmic stimulation is a therapeutic application of pulsed rhythmic beats or music as an external sensory stimulation (Thaut and Hoemberg, 2014). To patients with PD, the external cues may further increase activity in the putamen, a subcortical structure responsible for facilitating movement, and thus compensate for the lack of dopaminergic stimulation (Nombela et al., 2013). This benefit includes not only the improvement of general motor performance but also the ability to generate complex coordinated movement sequences (Thaut and Abiru, 2010). Second, the significant change in motor function may be attributed to extra motor learning. Multiple skill acquisitions being involved in intervention could promote the positive transfer of training, that is, a skill acquired in one circumstance enhances skill acquisition and problem-solving ability development in another circumstance (Krasnow and Wilmerding, 2016; Zou et al., 2021; Yu et al., 2022). This explanation is further supported by our findings that both the second and third likely best exercise forms (i.e., yoga and multimodal training) to general motor function are characterized by multiple movement tasks (Yeung et al., 2018; Yu et al., 2021).

In terms of mobility and balance performance, the network meta-analysis revealed that Nordic walking displayed significantly superior attainment, in comparison to all the other exercise modes. Using the Nordic poles to facilitate walking is a much more “conscious” way for movement production. The use of poles could act as tactile and proprioceptive cues providing the necessary triggers in PD to bypass the defective basal ganglia circuitries, which might be the direct reason for the observed improvement (Piek, 1998; van Eijkeren et al., 2008). Such external sensory cues have been demonstrated their usefulness in the struggle against impaired mobility in PD (van Wegen et al., 2006). Indirectly, allowing an adaptation to training with the use of poles could improve patients' fear of falling, and then have a higher level of compliance (Church et al., 2002; van Eijkeren et al., 2008). Similarly, aquatic training (i.e., the exercise mode that ranked second in mobility and balance to this study) also advocates a safer environment for training to reduce the fear of injuries (Gomes Neto et al., 2020). These inherent features may have led to enhanced efficacy of interventions.

Qigong and cycling emerged from the network meta-analysis as the most likely to improve manual dexterity based on the small effect sizes found in the current study (i.e., >0.2; Warner, 2012). What these two exercises have in common, in terms of movement features, is specific attention on the hands during practice. Cycling participants tend to put a part of the weight on their hands to maintain postural stability, while Qigong stresses hand postures during exercise (Zou et al., 2018, 2019). Experienced practitioners also described that they could feel “changes in skin temperature on the palms” during Qigong practice (Tse, 1996; Bertschinger, 2012). However, this conclusion was limited by the inconsistency of measurements being used, which only eight studies covering six exercise and two control treatments were included for the network meta-analysis in the current study. Hand dysfunction has been found to be strongly correlated with classical motor functions such as balance and trunk stability (Colakoglu et al., 2016). While a great number of studies investigated the relationship between exercise intervention and classic motor symptoms, little is known about the efficacy of exercise on hand function in PD. We would suggest that future exercise training studies include and report hand function along with other motor functions. This also suggests that a precise and standardized measurement tool is needed, as most of the included studies assessed hand dexterity *via* different tools.

Strengths of this study may include that a considerable sample size of patients with PD (*n* = 4,631), thus providing the power to detect statistically significant mean differences. Comprehensive considerations on the types of intervention strategies (*n* = 16) also strengthened the study, which covered 14 types of exercises. Only RCTs, the gold standard for clinical interventions, were included. This study also explored the medication state (during assessment) with a subgroup analysis that is regularly omitted in previous reviews.

The current study may also have several limitations. First, no statistical difference was found in the demographics (e.g., age, duration of PD, H&Y stage) on exercise modes, and confounders were hard to be fully controlled, which may explain part of the inconsistencies. However, this may increase the generalizability of the results from the study. Second, we were unable to evaluate the implementation level of interventions (e.g., training intensity and difficulty). As a result, the dose-response effects were not examined, so the dose-response effects were not able to investigate. Third, due to the effect that the follow-up data were rarely reported and there was inconsistent time of examinations, the current study only provided the data at the endpoint of interventions. Therefore, clinical changes after the interventions ended were not analyzed. Finally, evaluation of the efficacy of specific exercise modes was limited by the number of studies available and variability in reporting.

## 5. Conclusion

The current study provides valuable information for the clinical application of exercise in patients with PD. The analyses of the long-term efficacy of exercise have showed that exercise would slow the progression of motor function in PD. In addition, TUG might be a more sensitive indicator to reflect changes in motor function than UPDRS-motor scale and BBS. Furthermore, the findings of the current study suggest that dancing might be a superior exercise for general motor symptoms; Nordic walking may be the most efficient exercise to sustain mobility and balance, Qigong may have a significant benefit in improving manual dexterity. More research is needed to confirm these findings in an effort to optimize exercise protocols for PD. In summary, dancing, yoga, multimodal training, Nordic walking, aquatic training, exercise gaming, and Qigong are recommended exercises for sustaining motor function in PD.

## Data availability statement

The original contributions presented in the study are included in the article/Supplementary material, further inquiries can be directed to the corresponding author.

## Author contributions

MZ and FL generated the manuscript. DW and XB contributed to the data analysis. ZL helped to revise the manuscript. All authors contributed to the article and approved the submitted version.
