# Supplementary material for: Exercise sustains motor function in Parkinson's disease: Evidence from 109 randomized controlled trials on over 4,600 patients
Source: Front Aging Neurosci. 2023 Feb 14;15:1071803. doi: 10.3389/fnagi.2023.1071803 (PMC9971593; doi:10.3389/fnagi.2023.1071803)
Supplement: Supplementary file 1 [file Data_Sheet_1.PDF]

Supplementary Content

Searching strategy....2

Figure S1. PRISMA flowchart 3

Figure S2. The map of network meta-analysis for “ON state” UPDRS - motor scores 4

Figure S3. The results of pairwise comparisons for “OFF state” UPDRS - motor scores. 5

Figure S4. Funnel plots for each outcome: UPDRS - motor, balance, mobility, and manual dexterity....6

a. UPDRS - motor 6

b. Balance 7

c. Mobility 8

d. Manual dexterity 9

Figure S5. Cumulative ranking probability plots for UPDRS - motor scores 10

Table S1. Overall characteristics on exercise modes....11

Table S2. Intervention characteristics of included studies 12

Table S3. List of included studies....12

Table S4. Network meta-analysis consistency models for “ON state” UPDRS - motor scores....21

Searching strategy

[(Parkinson + Disease) OR (Idiopathic + Parkinson's + Disease) OR (Lewy + Body + Parkinson's + Disease) OR (Parkinson's + Disease, Idiopathic) OR (Parkinson's + Disease, Lewy + Body) OR (Parkinson + Disease, Idiopathic) OR (Parkinson's + Disease) OR (Idiopathic + Parkinson + Disease) OR (Lewy + Body + Parkinson + Disease) OR (Primary + Parkinsonism) OR (Parkinsonism, Primary) OR (Paralysis + Agitans)] AND [(Exercise*) OR (Physical activity) OR (aerobic*) OR (aquatic*) OR (water + based + exercise) OR (water + based + training) OR (swimming) OR (boxing) OR (cycling) OR (bike*) OR (bicycle*) OR (dancing) (virtual + reality + training) OR (exergame*) OR (functional + training) OR (functional + fitness) OR (Nordic + walking) OR (walking) OR (treadmill*) OR (Qigong) OR (strength + training) OR (resistance + training) OR (resistance + exercise) OR (stretching) OR (tai + chi) OR (yoga)] AND [(randomized + controlled + trial*) OR (controlled + clinical + trial*) OR (randomized) OR (placebo) OR (drug + therapy) OR (randomly) OR (trial*) OR (group*) NOT (animals)].

Figure S1. PRISMA flowchart

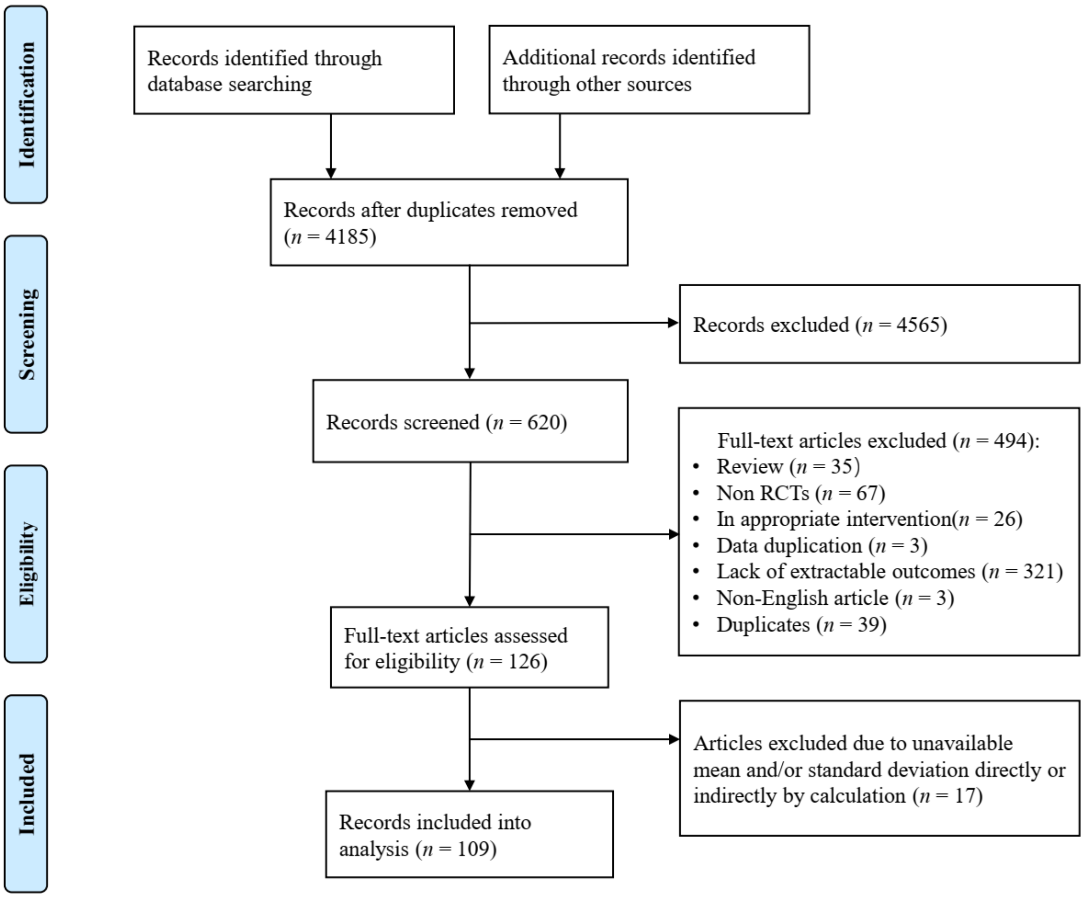

*PRISMA, Preferred Reporting Items for Systematic Reviews and Meta-Analysis; RCT, randomized controlled trial.

Figure S2. The map of network meta-analysis for "ON state" UPDRS-motor scores

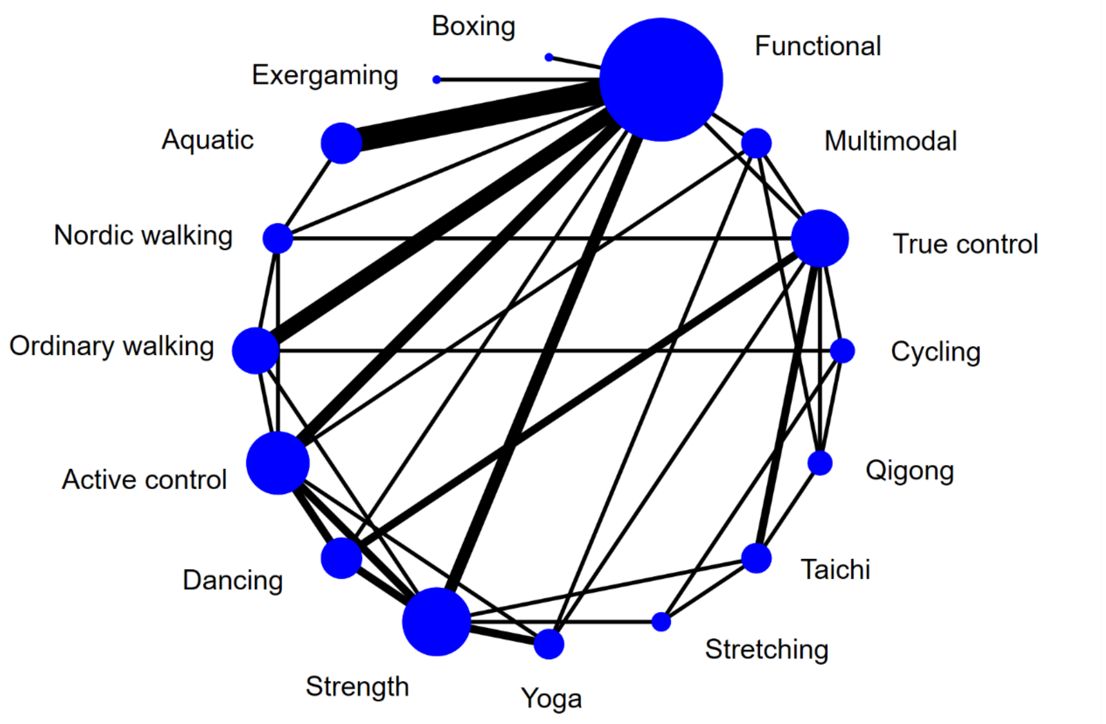

Figure S3. The results of pairwise comparisons for "OFF state" UPDRS-motor scores

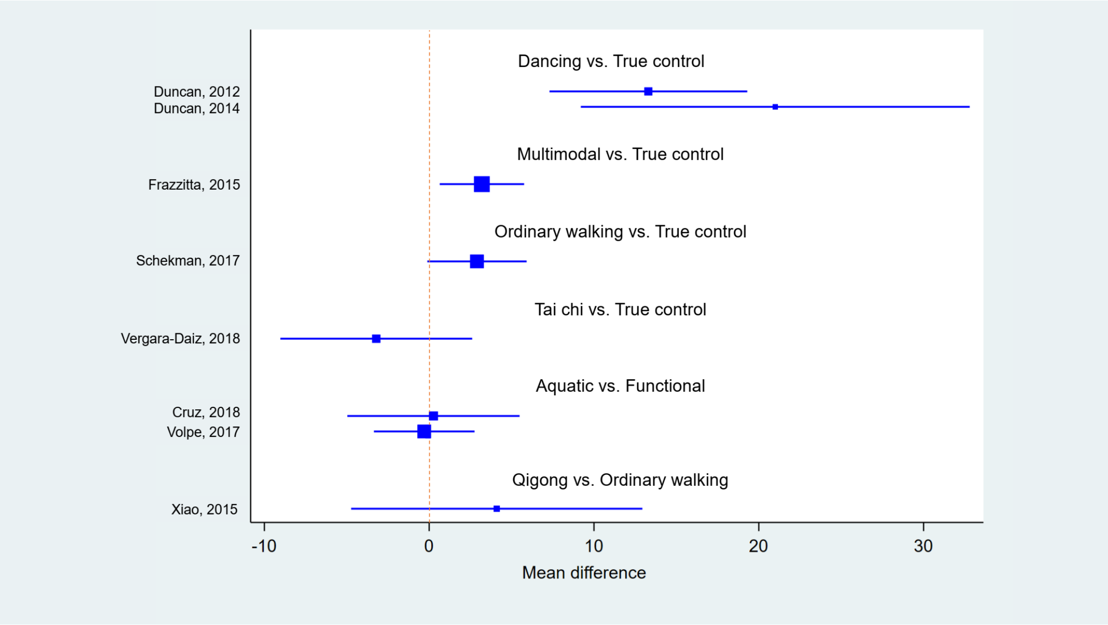

Figure S4. Funnel plots for each outcome: UPDRS-motor, balance, mobility, and manual dexterity

a. UPDRS - motor

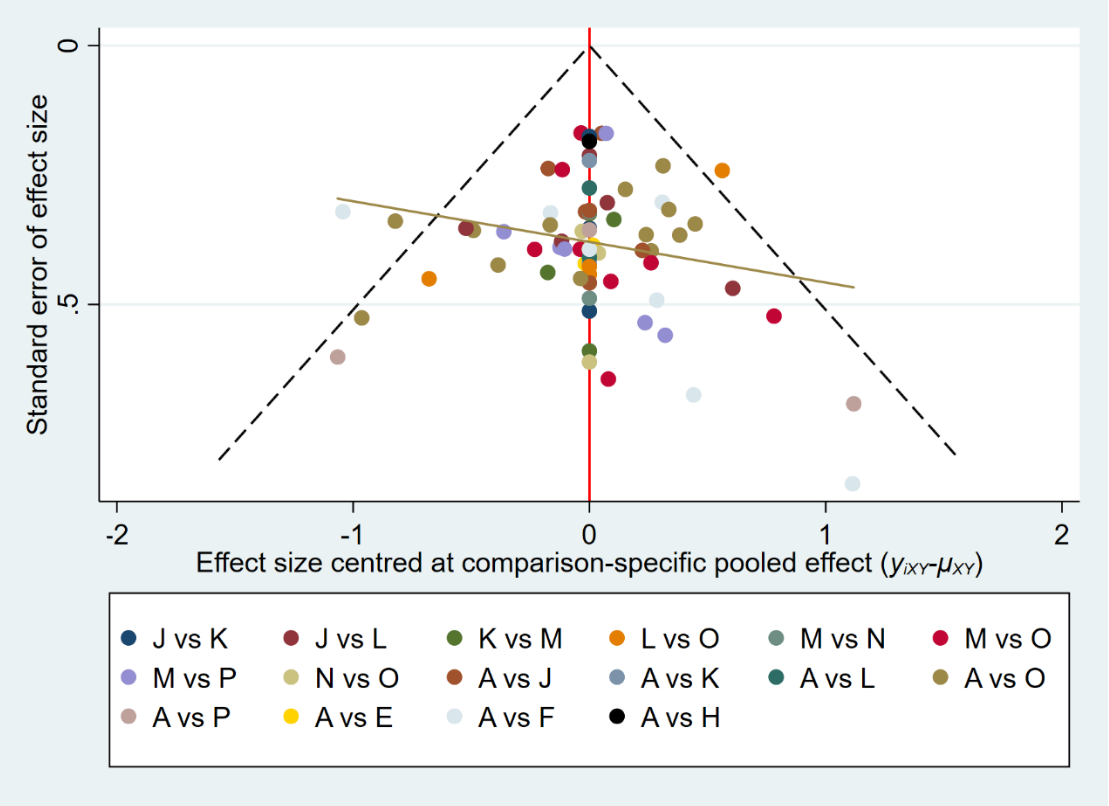

b. Balance

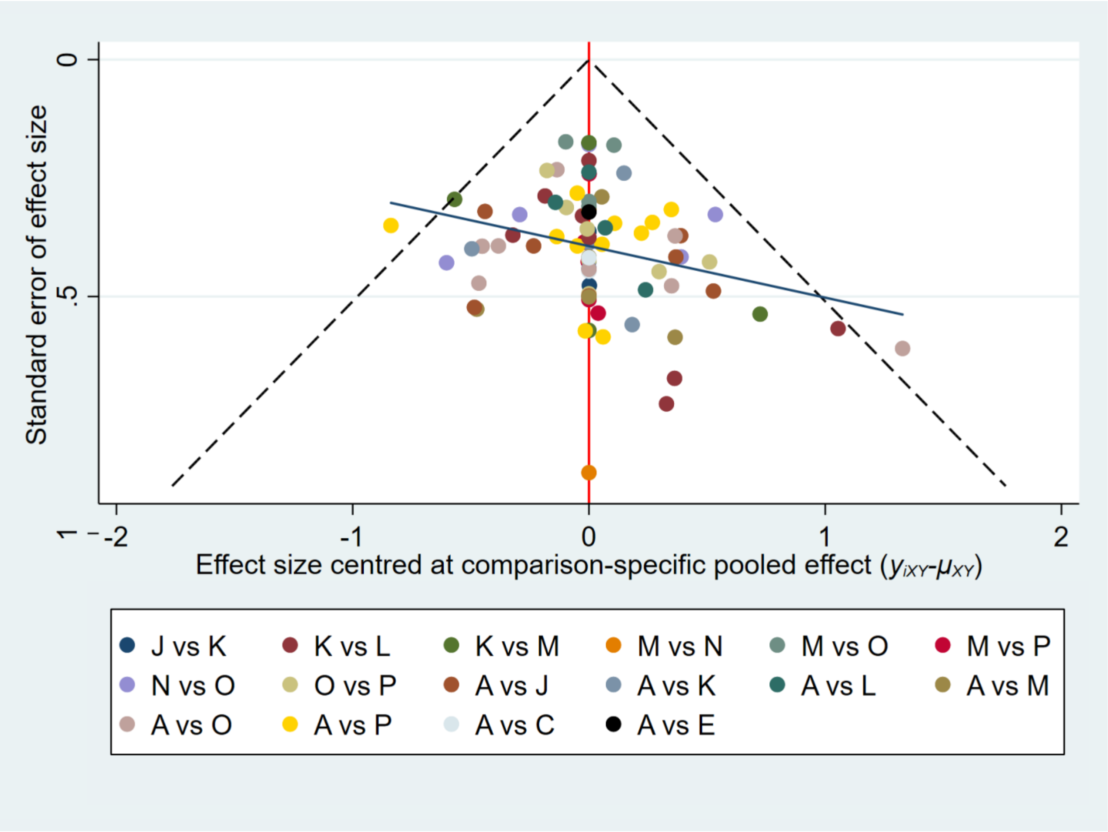
Standard error of effect size
0
.5
1
-2
-1
0
1
2
Effect size centred at comparison-specific pooled effect (y_{iXY}-μ_{XY})
J vs K
K vs L
K vs M
M vs N
M vs O
M vs P
N vs O
O vs P
A vs J
A vs K
A vs L
A vs M
A vs O
A vs P
A vs C
A vs E

c. Mobility

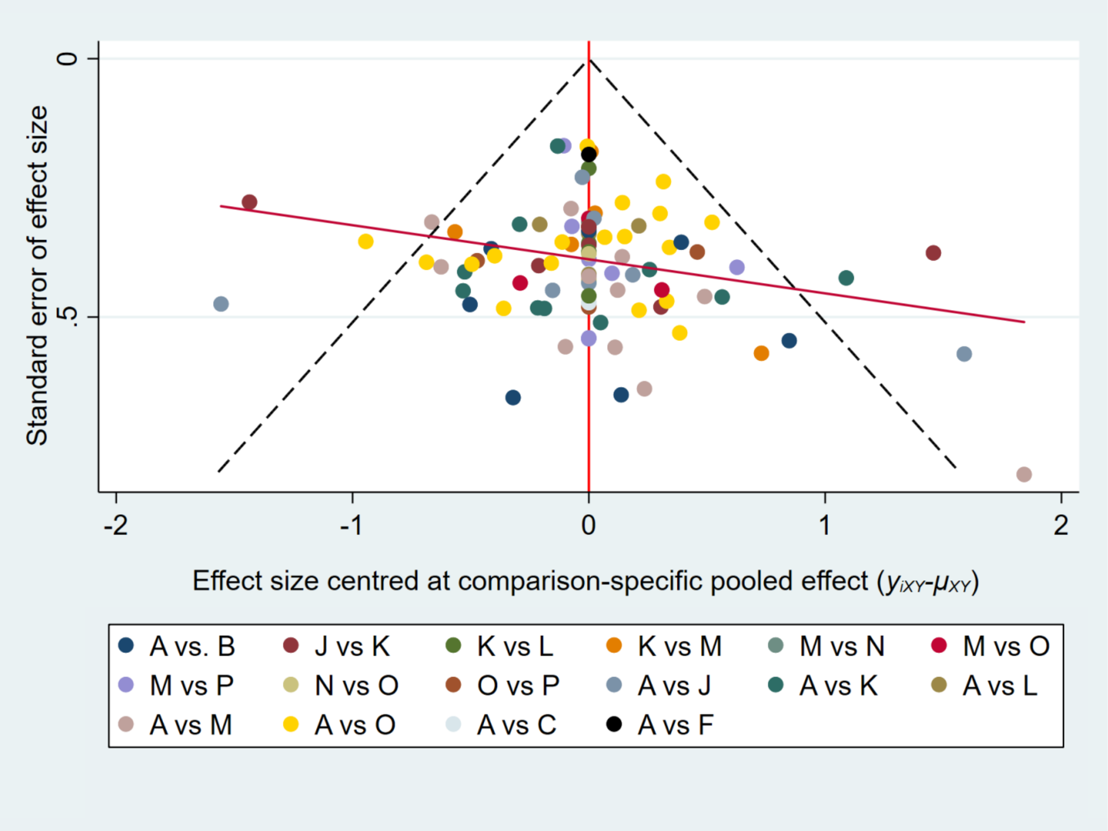

0
.5
Standard error of effect size
-2
-1
0
1
2
Effect size centred at comparison-specific pooled effect (y_{iXY}-μ_{XY})
A vs. B
J vs K
K vs L
K vs M
M vs N
M vs O
M vs P
N vs O
O vs P
A vs J
A vs K
A vs L
A vs M
A vs O
A vs C
A vs F

d. Manual dexterity

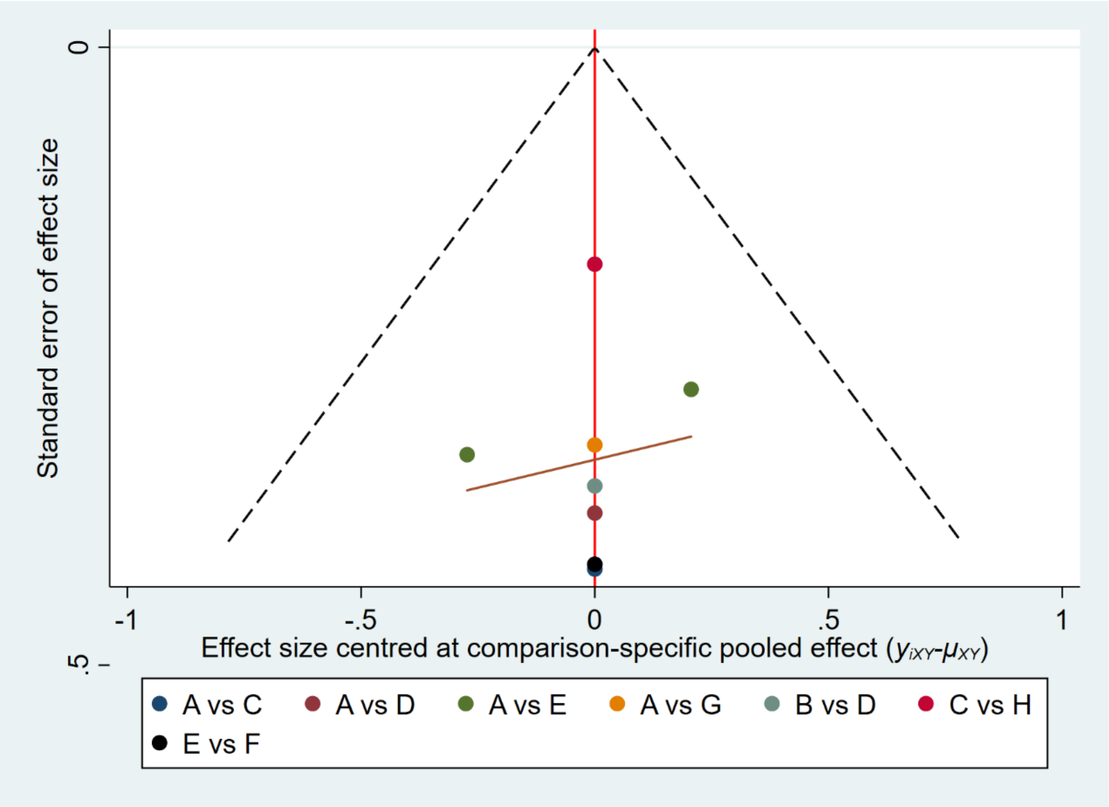

Figure S5. Cumulative ranking probability plots for UPDRS-motor scores

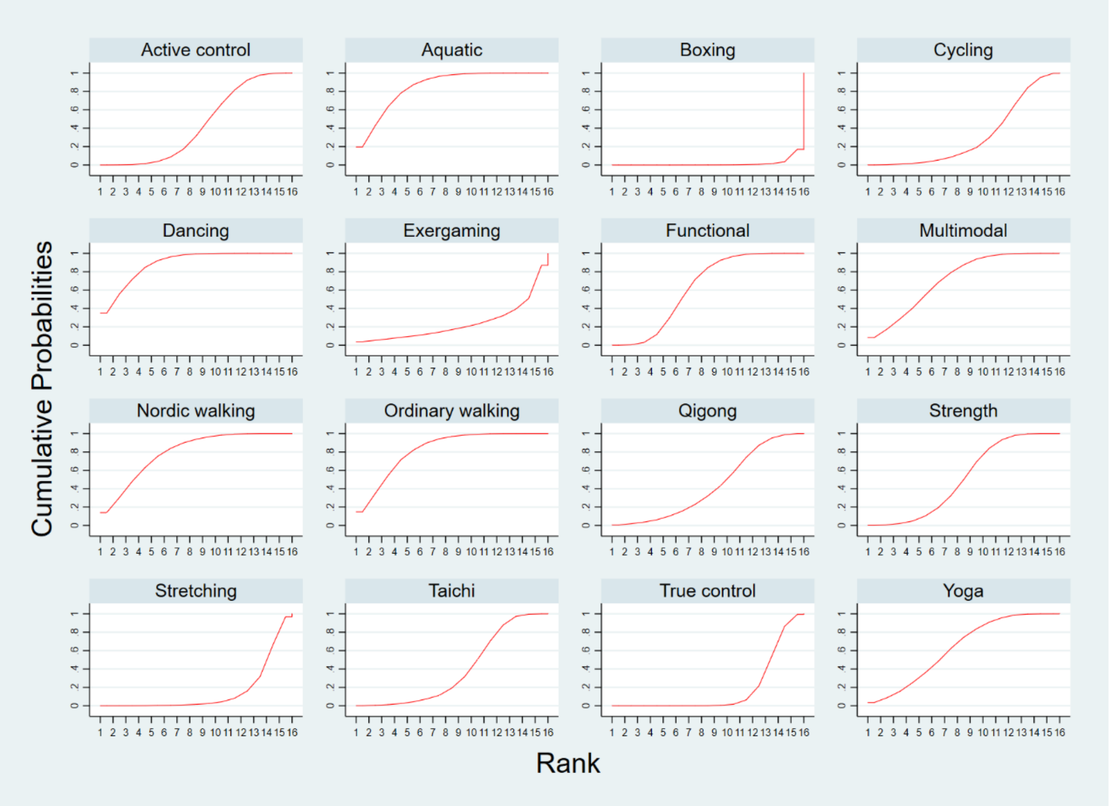

Table S1. Overall characteristics on exercise modes

	No. (%) of trials	No. of patients (*N*)	Mean age (*y*)	Mean PD year (*y*)	Mean H&Y stage
**Type**					
Aquatic	14 (6.0%)	187	67	6.9	2.5
Boxing	2 (0.9%)	37	66	4.5	2.3
Cycling	5 (2.1%)	140	65	6.7	2.3
Dancing	19 (8.1%)	331	67	6.9	2.2
Exergaming	12 (5.1%)	224	68	6.8	2.1
Functional	36 (15.4%)	709	66	6.5	2.3
Multimodal	15 (6.4%)	337	68	6.8	2.1
Nordic walking	6 (2.6%)	86	66	5.3	2.4
Qigong	6 (2.6%)	138	65	7.9	2.3
Strength	17 (7.3%)	376	66	6.9	2.2
Stretching	2 (0.9%)	130	69	6.5	2.2
Tai chi	12 (5.1%)	237	66	6.0	2.3
Ordinary walking	19 (8.1%)	393	67	5.2	2.3
Yoga	9 (3.9%)	163	69	6.4	2.2
**Control**					
Active control	13 (5.6%)	322	68	7.1	2.2
True control	45 (19.2%)	821	68	5.9	2.3
**Total**	234 (100%)	4631	67	7.1	2.3

Table S2. Intervention characteristics of included studies

Characteristics	No. (%) of trials ($N = 114$)
**Duration of intervention by weeks**	
≤ 4	16(14.0)
5 ~ 8	32(28.1)
9 ~ 12	40(35.1)
> 12	20(17.5)
Incomplete data	6(5.3)
**Duration of intervention by hours**	
< 15	36 (31.6)
16 ~ 30	46 (40.4)
31 ~ 45	12 (10.5)
46 ~ 60	9 (7.9)
> 60	7 (6.1)
Incomplete data	4 (3.5)
**Number of total intervention sessions**	
< 15	23 (20.2)
15 ~ 29	53 (46.5)
30 ~ 44	17 (14.9)
45 ~ 59	8 (7.0)
> 60	7 (6.1)
Incomplete data	6 (5.3)
**Number of sessions per week**	
1	9(7.9)
2	49(43.0)
3	29(25.4)
4	5(4.39)
≥ 5	13(14.0)
Incomplete data	6(5.3)
**Session duration (minutes)**	
≤ 15	1 (0.9)
16 ~ 30	9 (7.9)
31 ~ 45	22 (19.3)
46 ~ 60	67 (58.8)
> 60	11 (9.6)
Incomplete data	4 (3.5)

Note. 5 of the 109 included studies used different intervention duration on groups, so a total of 114 trials.

Table S3. List of included studies

Author	Year	Title	Source	DOI
Alessandro	2015	Comparison of strength training, aerobic training, and additional physical therapy as supplementary treatments for Parkinson's disease: pilot study	Clinical Interventions in Aging	10.2147/CIA.S68779
Allen	2010	The effects of an exercise program on fall risk factors in people with Parkinson's disease: A randomized controlled trial	Movement Disorders	10.1002/mds.23082
Allen	2017	An interactive videogame for arm and hand exercise in people with Parkinson's disease: A randomized controlled trial	Parkinsonism & Related Disorders	10.1016/j.parkreldis.2017.05.011
Amano	2013	The effect of Tai Chi exercise on gait initiation and gait performance in persons with Parkinson's disease	Parkinsonism & Related Disorders	10.1016/j.parkreldis.2013.06.007
Arcolin	2015	Intensive cycle ergometer training improves gait speed and endurance in patients with Parkinson’s disease: A comparison with treadmill training	Restorative Neurology and Neuroscience	10.3233/RNN-150506
Ashburn	2006	A randomized controlled trial of a home-based exercise program to reduce the risk of falling among people with Parkinson's disease	Journal of Neurology, Neurosurgery & Psychiatry	10.1136/jnnp.2006.099333
Bang	2017	Effects of an intensive Nordic walking intervention on the balance function and walking ability of individuals with Parkinson’s disease: a randomized controlled pilot trial	Aging Clinical and Experimental Research	10.1007/s40520-016-0648-9
Barbalho	2019	Effects of Low-Volume Resistance Training on Muscle Strength and Functionality of People with Parkinson’s Disease	International Journal of Exercise Science	N/A
Bega	2016	Yoga Versus Resistance Training in Mild to Moderate Severity Parkinson’s Disease: A 12-Week Pilot Study	Journal of Yoga & Physical Therapy	10.4172/2157-7595.1000222
Burini	2006	A randomised controlled cross-over trial of aerobic training A versus Qigong in advanced Parkinson’s disease	Europa Medicophysica	N/A
Cakit	2007	The effects of incremental speed-dependent treadmill training on postural instability and fear of falling in Parkinson's disease	Clinical Rehabilitation	10.1177/0269215507077269
Canning	2012	Home-based treadmill training for individuals with Parkinson’s disease: a randomized controlled pilot trial	Clinical Rehabilitation	10.1177/0269215511432652

Cherup	2021	Yoga Meditation Enhances Proprioception and Balance in Individuals Diagnosed with Parkinson's Disease	Perceptual and Motor Skills	10.1177/003151 2520945085
Cheung	2018	Effects of yoga on oxidative stress, motor function, and non-motor symptoms in Parkinson's disease: a pilot randomized controlled trial	Pilot and Feasibility Studies	10.1186/s40814-018-0355-8
Choi	2016	Effects of therapeutic Tai chi on functional fitness and activities of daily living in patients with Parkinson disease	Journal of Exercise Rehabilitation	10.12965/jer.163 2654.327
Choi	2013	Therapeutic Effects of Tai Chi in Patients with Parkinson's Disease	ISRN Neurology	10.1155/2013/54 8240
Colgrove	2012	Effect of Yoga on Motor Function in People with Parkinson's Disease: A Randomized, Controlled Pilot Study	Journal of Yoga & Physical Therapy	10.4172/2157-7595.1000112
Combs	2013	Community-based group exercise for persons with Parkinson disease: A randomized controlled trial	Neuro Rehabilitation	10.3233/NRE-130828
Corcos	2013	A two-year randomized controlled trial of progressive resistance exercise for Parkinson's disease: Progressive Resistance Exercise in PD	Movement Disorders	10.1002/mds.253 80
Cugusi	2015	Effects of a Nordic Walking program on motor and non-motor symptoms, functional performance and body composition in patients with Parkinson's disease	Neuro Rehabilitation	10.3233/NRE-151257
Dashtipour	2015	Effect of Exercise on Motor and Nonmotor Symptoms of Parkinson's Disease	Parkinson's Disease	10.1155/2015/58 6378
Dibble	2015	Exercise and Medication Effects on Persons with Parkinson Disease Across the Domains of Disability: A Randomized Clinical Trial	Journal of Neurologic Physical Therapy	10.1097/NPT.00 00000000000008 6
Duncan	2012	Randomized Controlled Trial of Community-Based Dancing to Modify Disease Progression in Parkinson Disease	Neurorehabilitation and Neural Repair	10.1177/154596 8311421614
Duncan	2014	Are the Effects of Community-Based Dance on Parkinson Disease Severity, Balance, and Functional Mobility Reduced with Time? A 2-Year Prospective Pilot Study	The Journal of Alternative and Complementary Medicine	10.1089/acm.201 2.0774
Ebersbach	2010	Comparing exercise in Parkinson's disease-the Berlin BIG Study: Exercise in Parkinson's Disease	Movement Disorders	10.1002/mds.232 12

Fernández-González	2019	Leap motion-controlled video game-based therapy for upper limb rehabilitation in patients with Parkinson’s disease: a feasibility study	Journal of NeuroEngineering and Rehabilitation	10.1186/s12984-019-0593-x
Ferraz	2018	The Effects of Functional Training, Bicycle Exercise, and Exergaming on Walking Capacity of Elderly Patients with Parkinson Disease: A Pilot Randomized Controlled Single-blinded Trial	Archives of Physical Medicine and Rehabilitation	10.1016/j.apmr.2017.12.014
Fisher	2008	The Effect of Exercise Training in Improving Motor Performance and Corticomotor Excitability in People with Early Parkinson's Disease	Archives of Physical Medicine and Rehabilitation	10.1016/j.apmr.2008.01.013
Franzoni	2018	A 9-Week Nordic and Free Walking Improve Postural Balance in Parkinson’s Disease	Sports Medicine International Open	10.1055/s-0043-124757
Frazzitta	2012	Effectiveness of Intensive Inpatient Rehabilitation Treatment on Disease Progression in Parkinsonian Patients: A Randomized Controlled Trial With 1-Year Follow-up	Neurorehabilitation and Neural Repair	10.1177/1545968311416990
Frazzitta	2015	Intensive Rehabilitation Treatment in Early Parkinson’s Disease: A Randomized Pilot Study With a 2-Year Follow-up	Neurorehabilitation and Neural Repair	10.1177/1545968314542981
Frisaldi	2021	Effectiveness of a dance-physiotherapy combined intervention in Parkinson’s disease: a randomized controlled pilot trial	Neurological Sciences	10.1007/s10072-021-05171-9
Gandolfi	2017	Virtual Reality Telerehabilitation for Postural Instability in Parkinson’s Disease: A Multicenter, Single-Blind, Randomized, Controlled Trial	BioMed Research International	10.1155/2017/7962826
Gao	2014	Effects of Tai Chi on balance and fall prevention in Parkinson’s disease: a randomized controlled trial	Clinical Rehabilitation	10.1177/0269215514521044
Goodwin	2011	An exercise intervention to prevent falls in people with Parkinson's disease: a pragmatic randomised controlled trial	Journal of Neurology, Neurosurgery & Psychiatry	10.1136/jnnp-2011-300919
Hackney	2008	Tai Chi improves balance and mobility in people with Parkinson disease	Gait & Posture	10.1016/j.gaitpost.2008.02.005
Hackney	2010	Effects of Dance on Gait and Balance in Parkinson’s Disease: A Comparison of Partnered and Non-partnered Dance Movement	Neurorehabilitation and Neural Repair	10.1177/1545968309353329

Hackney	2007	Effects of Tango on Functional Mobility in Parkinson's Disease: A Preliminary Study	Journal of Neurologic Physical Therapy	10.1097/NPT.0b013e31815ce78b
Hackney	2009	Effects of dance on movement control in Parkinson's disease: A comparison of Argentine tango and American ballroom	Journal of Rehabilitation Medicine	10.2340/16501977-0362
Helgerud	2020	Maximal strength training in patients with Parkinson's disease: impact on efferent neural drive, force-generating capacity, and functional performance	Journal of Applied Physiology	10.1152/japplphysiol.00208.2020
Khuzema	2020	Effect of home-based Tai Chi, Yoga or conventional balance exercise on functional balance and mobility among persons with idiopathic Parkinson's disease: An experimental study	Hong Kong Physiotherapy Journal	10.1142/S1013702520500055
Kunkel	2017	A randomized controlled feasibility trial exploring partnered ballroom dancing for people with Parkinson's disease	Clinical Rehabilitation	10.1177/0269215517694930
Kwok	2019	Effects of Mindfulness Yoga vs Stretching and Resistance Training Exercises on Anxiety and Depression for People with Parkinson Disease: A Randomized Clinical Trial	JAMA Neurology	10.1001/jamaneurol.2019.0534
Langer	2021	A randomised controlled trial on effectiveness and feasibility of sport climbing in Parkinson's disease	npj Parkinson's Disease	10.1038/s41531-021-00193-8
Leal	2019	Low-volume resistance training improves the functional capacity of older individuals with Parkinson's disease	Geriatrics & Gerontology International	10.1111/ggi.13682
Lee	2018	Turo (Qi Dance) Program for Parkinson's Disease Patients: Randomized, Assessor Blind, Waiting-List Control, Partial Crossover Study	EXPLORE	10.1016/j.explore.2017.11.002
Lee	2015	Effect of virtual reality dance exercise on the balance, activities of daily living, and depressive disorder status of Parkinson's disease patients	Journal of Physical Therapy Science	10.1589/jpts.27.145
Li	2012	Tai Chi and Postural Stability in Patients with Parkinson's Disease	New England Journal of Medicine	10.1056/NEJMoa1107911
Liao	2015	Virtual Reality–Based Training to Improve Obstacle-Crossing Performance and Dynamic Balance in Patients with Parkinson's Disease	Neurorehabilitation and Neural Repair	10.1177/1545968314562111
Lima	2019	Resistance training reduces depressive symptoms in elderly people with Parkinson disease: A controlled randomized study	Scandinavian Journal of Medicine & Science in Sports	10.1111/sms.13528

Liu	2016	Effects of Health Qigong Exercises on Relieving Symptoms of Parkinson’s Disease	Evidence-Based Complementary and Alternative Medicine	10.1155/2016/5935782
McKee	2013	The Effects of Adapted Tango on Spatial Cognition and Disease Severity in Parkinson's Disease	Journal of Motor Behavior	10.1080/00222895.2013.834288
Michels	2018	“Dance Therapy” as a psychotherapeutic movement intervention in Parkinson’s disease	Complementary Therapies in Medicine	10.1016/j.ctim.2018.07.005
Miyai	2000	Treadmill training with body weight support: Its effect on Parkinson's disease	Archives of Physical Medicine and Rehabilitation	10.1053/apmr.2000.4439
Miyai	2002	Long-term effect of body weight–supported treadmill training in Parkinson's disease: A randomized controlled trial	Archives of Physical Medicine and Rehabilitation	10.1053/apmr.2002.34603
Monteiro	2017	Effects of Nordic walking training on functional parameters in Parkinson's disease: a randomized controlled clinical trial	Scandinavian Journal of Medicine & Science in Sports	10.1111/sms.12652
Moon	2020	Can Qigong improve non-motor symptoms in people with Parkinson's disease - A pilot randomized controlled trial?	Complementary Therapies in Clinical Practice	10.1016/j.ctcp.2020.101169
Morris	2015	A Randomized Controlled Trial to Reduce Falls in People With Parkinson’s Disease	Neurorehabilitation and Neural Repair	10.1177/1545968314565511
Morris	2017	A home program of strength training, movement strategy training and education did not prevent falls in people with Parkinson’s disease: a randomised trial	Journal of Physiotherapy	10.1016/j.jphys.2017.02.015
Myers	2020	Yoga Improves Balance and Low-Back Pain, but Not Anxiety, in People with Parkinson’s Disease	International Journal of Yoga Therapy	10.17761/2020-D-18-00028
Nadeau	2014	Effects of 24 wk of Treadmill Training on Gait Performance in Parkinson’s Disease	Medicine & Science in Sports & Exercise	10.1249/MSS.00000000000000144

Ni	2016	Controlled pilot study of the effects of power yoga in Parkinson's disease	Complementary Therapies in Medicine	10.1016/j.ctim.2016.01.007
Ni	2016	Comparative Effect of Power Training and High-Speed Yoga on Motor Function in Older Patients with Parkinson Disease	Archives of Physical Medicine and Rehabilitation	10.1016/j.apmr.2015.10.095
Passos-Monteiro	2020	Nordic Walking and Free Walking Improve the Quality of Life, Cognitive Function, and Depressive Symptoms in Individuals with Parkinson's Disease: A Randomized Clinical Trial	Journal of Functional Morphology and Kinesiology	10.3390/jfmk5040082
Paul	2014	Leg muscle power is enhanced by training in people with Parkinson's disease: a randomized controlled trial	Clinical Rehabilitation	10.1177/0269215513507462
Picelli	2016	Effects of treadmill training on cognitive and motor features of patients with mild to moderate Parkinson's disease: a pilot, single-blind, randomized controlled trial	Functional Neurology	10.11138/FNeur/2016.31.1.025
Poier	2019	A Randomized Controlled Trial to Investigate the Impact of Tango Argentina versus Tai Chi on Quality of Life in Patients with Parkinson Disease: A Short Report	Complementary Medicine Research	10.1159/000500070
Pompeu	2012	Effect of Nintendo Wii™-based motor and cognitive training on activities of daily living in patients with Parkinson's disease: A randomised clinical trial	Physiotherapy	10.1016/j.physio.2012.06.004
Protas	2005	Gait and step training to reduce falls in Parkinson's disease	Neuro Rehabilitation	10.3233/NRE-2005-20305
Qutubuddin	2013	Parkinson's Disease and Forced Exercise: A Preliminary Study	Rehabilitation Research and Practice	10.1155/2013/375267
Ribas	2017	Effectiveness of exergaming in improving functional balance, fatigue and quality of life in Parkinson's disease: A pilot randomized controlled trial	Parkinsonism & Related Disorders	10.1016/j.parkreldis.2017.02.006
Rios Romenets	2015	Tango for treatment of motor and non-motor manifestations in Parkinson's disease: A randomized control study	Complementary Therapies in Medicine	10.1016/j.ctim.2015.01.015
Rocha	2018	Dance therapy for Parkinson's disease: A randomised feasibility trial	International Journal of Therapy and Rehabilitation	10.12968/ijtr.2018.25.2.64

Ryan	2020	Interval, Active-Assisted Cycling Improves Motor Function but Does Not Alter Balance in Parkinson’s Disease	Dissertation	N/A
Sage	2009	Symptom and gait changes after sensory attention focused exercise vs aerobic training in Parkinson's disease	Movement Disorders	10.1002/mds.22469
Sangarapillai	2021	Boxing vs Sensory Exercise for Parkinson’s Disease: A Double-Blinded Randomized Controlled Trial	Neurorehabilitation and Neural Repair	10.1177/15459683211023197
Schenkman	2012	Exercise for People in Early- or Mid- Stage Parkinson Disease: A 16-Month Randomized Controlled Trial	Physical Therapy	10.2522/ptj.20110472
Schenkman	2018	Effect of High-Intensity Treadmill Exercise on Motor Symptoms in Patients With De Novo Parkinson Disease: A Phase 2 Randomized Clinical Trial	JAMA Neurology	10.1001/jamaneurol.2017.3517
Schilling	2010	Effects of Moderate-Volume, High-Load Lower-Body Resistance Training on Strength and Function in Persons with Parkinson's Disease: A Pilot Study	Parkinson's Disease	10.4061/2010/824734
Schlenstedt	2015	Resistance versus Balance Training to Improve Postural Control in Parkinson's Disease: A Randomized Rater Blinded Controlled Study	PLOS ONE	10.1371/journal.pone.0140584
Schmitz-Hübsch	2006	Qigong exercise for the symptoms of Parkinson's disease: A randomized, controlled pilot study: Qigong in PD	Movement Disorders	10.1002/mds.20705
Shanahan	2017	Dancing for Parkinson Disease: A Randomized Trial of Irish Set Dancing Compared with Usual Care	Archives of Physical Medicine and Rehabilitation	10.1016/j.apmr.2017.02.017
Shen	2014	Balance and Gait Training with Augmented Feedback Improves Balance Confidence in People with Parkinson’s Disease: A Randomized Controlled Trial	Neurorehabilitation and Neural Repair	10.1177/1545968313517752
Shih	2016	Effects of a balance-based exergaming intervention using the Kinect sensor on posture stability in individuals with Parkinson’s disease: a single-blinded randomized controlled trial	Journal of Neuro Engineering and Rehabilitation	10.1186/s12984-016-0185-y
Shinichi Amano	2013	Tai Chi Exercise to Improve Non-Motor Symptoms of Parkinson’s Disease	Journal of Yoga & Physical Therapy	10.4172/2157-7595.1000137
Shulman	2013	Randomized Clinical Trial of 3 Types of Physical Exercise for Patients with Parkinson Disease	JAMA Neurology	10.1001/jamaneurol.2013.646

Silva-Batista	2016	Resistance Training with Instability for Patients with Parkinson’s Disease	Medicine & Science in Sports & Exercise	10.1249/MSS.000000000000000945
Silva-Batista	2017	Instability Resistance Training Improves Neuromuscular Outcome in Parkinson's Disease	Medicine & Science in Sports & Exercise	10.1249/MSS.000000000000001159
Soke	2021	Task-oriented circuit training combined with aerobic training improves motor performance and balance in people with Parkinson′s Disease	Acta Neurological Belgica	10.1007/s13760-019-01247-8
Solla	2019	Sardinian Folk Dance for Individuals with Parkinson's Disease: A Randomized Controlled Pilot Trial	The Journal of Alternative and Complementary Medicine	10.1089/acm.2018.0413
Song	2018	Home-based step training using videogame technology in people with Parkinson’s disease: a single-blinded randomised controlled trial	Clinical Rehabilitation	10.1177/0269215517721593
Szefler-Derela	2020	Effectiveness of 6-Week Nordic Walking Training on Functional Performance, Gait Quality, and Quality of Life in Parkinson’s Disease	Medicina	10.3390/medicina56070356
Tollár	2018	A High-Intensity Multicomponent Agility Intervention Improves Parkinson Patients’ Clinical and Motor Symptoms	Archives of Physical Medicine and Rehabilitation	10.1016/j.apmr.2018.05.007
van der Kolk	2019	Effectiveness of home-based and remotely supervised aerobic exercise in Parkinson's disease: a double-blind, randomised controlled trial	The Lancet Neurology	10.1016/S1474-4422(19)30285-6
Vergara-Diaz	2018	Tai Chi for Reducing Dual-task Gait Variability, a Potential Mediator of Fall Risk in Parkinson’s Disease: A Pilot Randomized Controlled Trial	Global Advances in Health and Medicine	10.1177/2164956118775385
Vieira de Moraes Filho	2020	Progressive Resistance Training Improves Bradykinesia, Motor Symptoms and Functional Performance in Patients with Parkinson’s Disease	Clinical Interventions in Aging	10.2147/CIA.S231359
Volpe	2013	A comparison of Irish set dancing and exercises for people with Parkinson’s disease: A phase II feasibility study	BMC Geriatrics	10.1186/1471-2318-13-54
Walter	2019	Changes in Nonmotor Symptoms Following an 8-Week Yoga Intervention for People with Parkinson's Disease	International Journal of Yoga Therapy	10.17761/2019-00025

Wróblewska	2019	The Therapeutic Effect of Nordic Walking on Freezing of Gait in Parkinson's Disease: A Pilot Study	Parkinson's Disease	10.1155/2019/3846279
Xiao	2016	Effect of health Baduanjin Qigong for mild to moderate Parkinson's disease: Health qigong Baduanjin for PD patients	Geriatrics & Gerontology International	10.1111/ggi.12571
Yang	2016	Home-based virtual reality balance training and conventional balance training in Parkinson's disease: A randomized controlled trial	Journal of the Formosan Medical Association	10.1016/j.jfma.2015.07.012
Yuan	2020	Effects of interactive video-game–based exercise on balance in older adults with mild-to-moderate Parkinson's disease	Journal of NeuroEngineering and Rehabilitation	10.1186/s12984-020-00725-y
Zhang	2015	Effects of Tai Chi and Multimodal Exercise Training on Movement and Balance Function in Mild to Moderate Idiopathic Parkinson Disease	American Journal of Physical Medicine & Rehabilitation	10.1097/PHM.0000000000000351

Table S4. Network meta-analysis consistency models for “ON state” UPDRS - motor scores

Exercise mode	Comparison to 'true control''		Likelihood (%) of being…		SUCRA (%)
	Pooled SMD (95% CI)	P value	Best	Worst	
True control	–	–	0.0	0.6	18
Active control	3.20 (-0.54 to 6.95)	0.094	0.0	0.0	43
Aquatic	7.12 (2.93 to 11.31)	**0.001**	19.7	0.0	85
Boxing	-8.78(-17.17 to -0.38)	**0.040**	0.0	82.9	2
Cycling	1.62 (-2.77 to 6.00)	0.470	0.1	0.3	32
Dancing	7.54 (3.77 to 11.31)	**<0.001**	34.8	0.0	89
Exergaming	-1.58 (-12.94 to 9.78)	0.785	3.9	13.1	24
Functional	5.02 (1.56 to 8.49)	**0.005**	0.1	0.0	63
Multimodal	5.66 (2.00 to 9.32)	**0.002**	8.4	0.0	72
Nordic walking	6.56 (2.14 to 10.97)	**0.004**	14.0	0.0	80
Ordinary walking	6.80 (2.66 to 10.95)	**0.001**	14.8	0.0	82
Qigong	2.91 (-1.26 to 7.09)	0.171	0.6	0.0	43
Strength	3.83 (0.32 to 7.34)	**0.033**	0.1	0.0	51
Stretching	-0.94 (-5.72 to 3.83)	0.699	0.0	3.1	15
Taichi	2.53 (-0.92 to 5.98)	0.150	0.1	0.0	39
Yoga	4.88 (1.28 to 8.49)	**0.008**	3.5	0.0	63
